# The roles of endoglin gene in cerebrovascular diseases

**DOI:** 10.20517/2347-8659.2017.18

**Published:** 2017-10-17

**Authors:** Wan Zhu, Li Ma, Rui Zhang, Hua Su

**Affiliations:** 1 Center for Cerebrovascular Research, Department of Anesthesia and Perioperative Care, University of California, San Francisco, CA 94143, USA; 2 Department of Neurosurgery, Beijing Tiantan Hospital, Capital Medical University, Beijing 100050, China

**Keywords:** Endoglin, cerebrovascular disease, stroke, angiogenesis

## Abstract

Endoglin (ENG, also known as CD105) is a transforming growth factor β (TGFβ) associated receptor and is required for both vasculogenesis and angiogenesis. Angiogenesis is important in the development of cerebral vasculature and in the pathogenesis of cerebral vascular diseases. ENG is an essential component of the endothelial nitric oxide synthase activation complex. Animal studies showed that ENG deficiency impairs stroke recovery. ENG deficiency also impairs the regulation of vascular tone, which contributes to the pathogenesis of brain arteriovenous malformation (bAVM) and vasospasm. In human, functional haploinsufficiency of *ENG* gene causes type I hereditary hemorrhagic telangiectasia (HHT1), an autosomal dominant disorder. Compared to normal population, HHT1 patients have a higher prevalence of AVM in multiple organs including the brain. Vessels in bAVM are fragile and tend to rupture, causing hemorrhagic stroke. High prevalence of pulmonary AVM in HHT1 patients are associated with a higher incidence of paradoxical embolism in the cerebral circulation causing ischemic brain injury. Therefore, HHT1 patients are at risk for both hemorrhagic and ischemic stroke. This review summarizes the possible mechanism of ENG in the pathogenesis of cerebrovascular diseases in experimental animal models and in patients.

## INTRODUCTION

In human, endoglin gene (*ENG*, or CD105) is located on chromosome 9q34.11. It is a type III transforming growth factor β (TGFβ) receptor interacting with TGFβRI (TGFβ receptor, type I) and/or TGFβRII (TGFβ receptor, type II)^[1]^. In the endothelium, ENG interacts with the activin receptor-like kinase 1 (ALK1 or ACVRL1), a type 1 TGFβR. ENG binds with TGFβ1 and TGFβ3 with high affinity in the presence of other TGFRs but not with TGFβ2^[1–4]^. ENG also binds to activin-A, bone morphogenetic protein 2 (BMP2) and BMP7^[1]^. Protein studies suggested that ENG plays an important role in modulating the TGFβ signaling pathway^[4]^.

*ENG* gene expresses in many cell types, including endothelial cells^[5, 6]^, activated monocytes and macrophages^[7]^, mesenchymal cells, fibroblasts^[8]^, and vascular smooth muscle cells [Table 1]^[9, 10]^. Animals studies have revealed that ENG may be dispensable during vasculogenesis, a process from which primary capillary plexus is formed; but ENG is required in angiogenesis, a process that remodels the primary endothelial network into a mature circulatory system^[11, 12]^. Immunohistochemical analysis showed that in normal human brain, ENG is expressed in the endothelial cells of brain vessels, as well as the endothelial and adventitial layers of leptomeningeal arteries [Table 1]^[13]^. ENG expression is upregulated in endothelial cells during wound healing and tumor vascularization, and in inflammatory tissues and developing embryos^[1, 14, 15]^, indicating that ENG is an endothelial proliferation marker^[16, 17]^.

Ischemic stroke is caused by occlusion of a cerebral artery. After ischemic stroke, blood supply to the affected brain tissue is reduced, which leads to oxygen deprivation to brain cells. Ischemia induces a significant increase in microvascular density, a sign of angiogenesis, in the penumbra of the cerebral infarct^[18]^. The degree of increased vessel-density in the ischemic penumbra is positively correlated with the survival rate of stroke patients^[19]^. In addition, increased angiogenesis was associated improvement of functional outcome in both animal models and stroke patients^[20–23]^.

Mutations in the *ENG* gene are associated with type 1 hereditary hemorrhagic telangiectasia (HHT)^[24]^, also known as Osler-Rendu-Weber Syndrome. HHT is an autosomal dominant disease. The clinical features of HHT patients are telangiectases in mucocutaneous membrane and arteriovenous malformation (AVM) in multiple organs, including the skin, liver, lung, intestine and brain. AVMs are abnormal vessels that shunt blood directly from arteries to veins^[25]^. Brain AVM (bAVM) tends to rupture, which can cause life-threatening intracranial hemorrhage and hemorrhagic stroke^[25]^. Hemorrhage from bAVM can also cause long-term disability. Elevated levels of angiogenic factors including vascular endothelial growth factor (VEGF) were found in sporadic bAVM patients^[26, 27]^. High levels of VEGF are also associated with increase of blood-brain barrier (BBB) permeability and bAVM hemorrhage^[27–29]^. Similarly, HHT patients that have a higher incidence of AVMs in multiple organs also have an increased level of plasma VEGF^[30]^. All of these evidence suggest that angiogenesis is involved in the pathogenesis of bAVM.

Since ENG plays an important role in the angiogenesis, in this review, we summarize the influences of ENG on endothelial function and the angiogenesis, as well as how ENG-deficiency contributes to the pathogenesis of cerebrovascular diseases, including ischemic stroke and intra-cranial hemorrhage, as well as cerebrovascular malformation, stenosis and occlusion.

## THE FUNCTION OF *ENG* GENE IN ANGIOGENESIS

To study the functional role of ENG in development, *Eng* gene knockout mice were generated^[11, 31]^. Homozygous deletion of *Eng* gene in mice causes embryonic death by E10.5–11.5^[11, 31]^. The endothelial cells derived from *ENG* deficient human embryonic stem cells failed to organize effectively into tubular structures *in vitro*^[12]^. VEGF induced vascular network was also reduced in the metatarsal bone of *Eng* heterozygous knockout (*Eng*^−/−^) mouse embryo^[12]^. Consistently, depletion or inhibition of *ENG* gene in human endothelial cells mitigated VEGF-induced angiogenesis^[12]^. These findings suggest that ENG is required for the differentiation and sprouting of endothelial tubes, which are important processes of angiogenesis.

ENG also mediates endothelial-mesenchymal communication during angiogenesis^[11, 32, 33]^. The recruitment of vascular smooth muscle cells and pericytes to newly formed vascular network is impaired in *Eng* deficient mouse embryos^[11]^.

## ENG DEFICIENCY IS AN IMPORTANT RISK FACTOR FOR BOTH HEMORRHAGIC AND ISCHEMIC STROKES

As mentioned in previous sections, ENG deficiency is associated with HHT1, a familial disease that has bAVM as one of its major phenotypes. Brain AVM contains abnormal vessels, that are prone to rupture, causing intracranial hemorrhage and hemorrhagic stroke. In addition, patients with ENG deficiency (HHT1) have a higher incidence of pulmonary AVM (PAVM), which is associated with a high incidence of paradoxical embolism in the cerebral circulation and ischemic brain injury^[34]^. To understand bAVM pathogenesis and to develop therapeutic strategies, many *Eng* deficient mouse models were generated. Using these animal models, we are able to elucidate bAVM pathogenesis and test new therapies.

Since homozygous deletion of *Eng* gene in mouse causes embryonic lethality^[11, 31]^, mice with heterozygous deletion of *Eng* (*Eng*^+/−^)^[31]^ are used to study the pathogenesis of HHT patients. *Eng*^+/−^ mice exhibit many phenotypes that resemble those of HHT1 patients, including mucocutaneous telangiectases, external bleeding, and AVMs in the liver, lung, brain and gastrointestine^[35]^. Enlarged cerebrovascular structure was found in some *Eng*^+/−^ mice with evidence of hemorrhage^[35]^. However, penetrance of bAVM in *Eng*^+/−^ mice is very low, only 7%^[35]^, suggesting that heterozygous *Eng* deletion alone is not sufficient to cause bAVM formation. In addition, the differences of the penetration of HHT phenotypes in 129/Ola and C57BL/6 *Eng*^+/−^ mice suggests that modifier genes are contributing to the severity and heterogeneity of AVMs in HHT patients^[35]^.

Based on clinical studies, we and others found that VEGF levels are increased in the plasma of HHT patients and in surgically resected sporadic human bAVM specimens^[26, 27, 30]^. The intensity of VEGF staining is also correlated with microvessel density in nasal mucosa from HHT patients^[36]^. Together, abnormally high level of VEGF appears to be a fundamental part of AVM pathophysiology^[25, 30, 37–39]^. Based on these studies, we overexpressed VEGF in the mouse brain in conjunction with *Eng* deletion to generate bAVM models. In adult *Eng*^+/−^ mice, intra-brain injection of an adeno-associated viral vector expressing VEGF (AAV-VEGF) significantly increased the penetrance of cerebrovascular abnormality^[40]^. Almost all-adult *Eng*^+/−^ mice that received intra-brain injection of AAV-VEGF showed cerebrovascular abnormality^[40]^. However, unlike HHT1 patients, the vascular abnormality in *Eng*^+/−^ mice is at the capillary level.

Bone marrow-derived cells can infiltrate into the brain angiogenic region. We found that macrophages are the major bone marrow-derived cells recruited to the brain angiogenic foci^[41]^. Since the accumulation of bone marrow-derived macrophage in VEGF-induced brain angiogenic regions peaks earlier than the increase of vessel density, macrophages likely play a role in angiogenesis.

Using *Eng*^+/−^ mice, the influence of bone marrow derived cells in the development of bAVM has been studied. Transplantation of *Eng*^+/−^ bone marrow to wild type mice induced vascular dysplasia in the brain angiogenic regions, while transplantation of wild type bone marrow to *Eng*^+/−^ mice reduced the severity of vascular dysplasia in the brain angiogenic foci of *Eng*^+/−^ mice^[42]^. These data suggested that *Eng* gene mutation in bone marrow cells cause vascular dysplasia. Importantly, these data suggested that transplantation of normal bone marrow cells to bAVM patients could be a therapeutic option.

Although we were able to induce vascular dysplasia in the brain of *Eng*^+/−^ mice by overexpression of VEGF, arteriovenous shunts were not detected in these mice. Studies have shown that a combination of homozygous *Eng* inactivation and additional stimulations are needed for robust bAVM formation. Genetic studies also indicated that mutations of *Eng* modifier genes contribute to AVM formation^[43, 44]^.

To avoid embryonic death caused by homozygous *Eng* deletion, Allinson *et al*.^[45]^ generated an *Eng*-floxed (*Eng*^2f/2f^) mouse line that have the *Eng* gene exons 5–6 flanked by loxP sites. When Cre recombinase is present, the DNA sequence between the loxPsites will be deleted. To test whether homozygous *Eng* gene deletion plus angiogenic stimulation can initiate bAVM formation, an adeno virus expressing Cre recombinase (Ad-Cre) and AAV-VEGF were co-injected into the brain of *Eng*^2f/2f^ mice^[45, 46]^ to induce brain focal *Eng* gene deletion and angiogenesis. *Eng*^2f/2f^ mice with focal *Eng* gene deletion and angiogenic stimulation developed vascular dysplasia beyond the capillary level around the AAV-VEGF injection site eight weeks after the vector injection^[46]^. Robust bAVM have also developed in the AAV-VEGF induced brain angiogenic region in mice subjected to global *Eng* deletion at the age of 8 weeks old^[47]^. The bAVM phenotype in these mice highly resembled the phenotype of human bAVM^[47]^. Furthermore, *Eng*-null endothelial cells were found in the dysplastic vessels in the bAVM lesion^[47]^. Our studies are consistent with the studies on skin AVM development, and support the notion that an injury (angiogenic stimulation) is needed to induce bAVM.

*Eng*-deficient bAVM mouse models have been used to analyze the function of macrophages during bAVM pathogenesis. Although *Eng* deficiency has been shown to impair monocyte migration into injured tissue^[48–50]^, an increased number of bone barrow-derived macrophages and activated residential microglia was found in the bAVM lesion in mouse and human. Compared with normal macrophages, *Eng*-deficient macrophages show slower but more persistent infiltration into the brain angiogenic regions^[51]^. Delayed clearance of macrophages and persistent inflammation could exaggerate abnormal vascular phenotypes in bAVM^[51]^.

In addition to conditional knockout of *Eng* gene in adult mice, several cell-specific cre transgenic mouse lines have been used to induction of *Eng* deletion in specific cell-types. For example, the promoter of SM22α (smooth muscle actin) is used express cre in smooth muscle specifically. Although SM22α is predominantly expressed in smooth muscle cells in normal mice, Cre expression driven by the SM22α promoter in this transgenic mouse line was also found in other cell types, including endothelial cells^[52, 53]^. SM22α Cre; *Eng*^2f/2f^ mice have *Eng* gene deleted in the SM22α expressing cells during the embryonic developmental stage. We found 90% of SM22α Cre; *Eng*^2f/2f^ mice have spontaneously developed bAVM by 5 weeks of age and 50% of them died by 6 weeks of age^[47]^. BAVM lesions varied in size and location in these mice^[47]^. In addition to bAVMs, some of SM22α Cre; *Eng*^2f/2f^ mice also developed spinal and intestinal AVMs^[47]^. Because AVM develops in this mouse line spontaneously without exogenous VEGF stimulation, this model is an ideal model for testing new therapeutic strategies.

As mentioned above, *Eng* gene not only expresses in endothelial cells^[5, 6]^, but also expresses in activated monocytes/macrophages^[7]^, mesenchymal cells, fibroblasts^[8]^, and smooth muscle cells^[9, 10]^. Using transgenes that express cre specific cell-types, the *Eng* gene was conditionally deleted in different cell types in adult mice to determine which cell type is most crucial for AVM development^[54, 55]^. In SclCreER; *Eng*^2f/2f^ mice, which have *Eng* deleted in endothelial cells only, AVM formed in the skin around the ear wound and back wound^[54, 55]^. We found that bAVM develops in the brain angiogenic region in Pdgfb-iCreER; *Eng*^2f/2f^ mice that have *Eng* gene deletion specifically in endothelial cells. Myh11Cre ER-mediated *Eng* deletion in smooth muscle cells in adult mice did not cause AVM formation in the wound area of the skin^[54]^. Furthermore, LysMCre; *Eng*^2f/2f^ mice, which have *Eng* deleted in macrophages, did not develop AVM in any organ and in the brain angiogenic regions^[47]^. These studies indicate that *Eng* deletion in endothelial cells is essential for AVM formation in the brain and other organs^[47, 54]^.

*Eng*-deficient bAVM mouse models were valuable resources to test new therapies for the treatment of bAVM. Current treatments for bAVM are mostly invasive and associated with high morbidities and mortalities^[56]^. Since high VEGF level is involved in the pathogenesis of bAVM, we have tested the feasibility of use soluble FMS-like tyrosine kinase 1 (sFLT1) gene therapy to treat bAVM. Soluble FLT1 is an alternative transcript of FLT1 (or VEGFR1) containing only the extracellular domains of the receptor. Soluble FLT1 binds VEGF with high affinity in tissue, reduces VEGF signaling through its membrane-bound receptors, and thus inhibits VEGF-induced angiogenesis^[14]^. Systemic delivery of AAV9-sFLT1 into a bAVM mouse model that has *Eng* gene deleted globally reduced abnormal vessels in the bAVM region^[57]^. Intravenous delivery of AAV9-sFLT1 to SM22α Cre; *Eng*^2f/2f[57]^ mice that have spontaneously developed bAVMs reduced mortality and bAVM penetrance^[57]^. This study demonstrated that mouse models are important tools to test new therapies.

## HYPOXIA INDUCES ENG EXPRESSION

Hypoxia induces the expression of ENG in human and mouse brain microvascular endothelial cells^[16, 22, 58]^, which ameliorates endothelial cells apoptosis regardless of the presence or absence of TGFβ^[59]^. During hypoxia stress, TGFβ induces apoptosis of endothelial cells^[60, 61]^, but reduces the death of neurons^[62]^ and vascular smooth muscle cells^[61]^. Therefore, ENG is likely to antagonize the inhibitory effects of TGFβ1 on human vascular endothelial cells^[17, 63]^ and protect endothelial cells against apoptosis via TGFβ signaling or other independent pathways^[59]^.

Under hypoxia conditions, ENG expression increases in many cell-types, such as, human microvascular endothelial cells-1 (HMEC-1) and monocytic U-937 cell. It is likely that hypoxia regulates ENG expression through crosstalk of several signaling pathways^[58]^.

The transcriptional regulation of ENG expression under hypoxia condition was studied by a reporter assay using HeLa cells, and by the electrophoretic mobility shift assay (EMSA) using human umbilical cord vein endothelial cells (HUVECs). These assays confirmed the presence of a hypoxia response element (HRE) in the enhancer region of *ENG* gene^[64]^. Therefore, ENG expression can be induced by hypoxia through hypoxia-inducible factor-1 (HIF-1). A subsequent study suggested that hypoxic induction of Eng expression in bEnd.3 (a mouse brain endothelial cell line) cells was activated through ERK-p38 MAPK and JNK pathway^[16]^, instead of HIF-1^[58]^. In addition, Smad3 was reported to interact with HIF proteins to induce the overexpression of ENG^[64]^. Although these studies implicated links among multiple factors, further studies are required to better elucidate the exact transcriptional regulation of ENG expression under hypoxia conditions.

## ENG EXPRESSION IS UPREGULATED AFTER STROKE INJURY

Previous studies revealed that ENG was highly expressed in the penumbra region of human stroke lesion, where an increase of angiogenesis was found^[22]^. However, it was not clear at that time whether the angiogenesis was beneficial. In acute ischemic stroke patients, there is a robust mobilization of immature hematopoietic cells, colony-forming cells and long-term culture initiating cells^[65]^. It has been suggested that the degree of immature hematopoietic cell mobilization is directly correlated with the recovery of neurological function^[66, 67]^. An increase of ENG positive micro-particles including exosomes and shedding vesicles, which are small vesicles released by specific cells (endothelial or MSC)^[68]^, were detected in patients’ sera collected 3 days after stroke compared to that of healthy people^[69]^. Certain types of ENG positive micro-particles increased further in stroke patients with severe disability. The ENG positive micro-particles decreased gradually after the initial increases^[69]^. The number of these circulating ENG positive micro-particles was positively correlated with the stroke severity, even after adjusting for other demographic and clinical variables, such as hypotension and other stroke comorbidities^[69]^. Similarly, ENG positive circulating micro-particles released from endothelial cells were also increased in patients with acute ischemic stroke. The increase of ENG positive cells was positively associated with the severity of neurological function at hospital admission, larger brain lesion volume and unfavorable functional outcome at hospital discharge^[70]^. The increased level of circulating ENG positive micro-particles after acute stroke may have been caused by either increased circulating cells as a self-repair response to stroke or a sign of increased apoptosis of circulating cells in response to hypoxic conditions^[69]^.

The role of ENG in stroke injury is complex, and is influenced by the local microenvironment. Constitutive expression of ENG enhances the TGFβ signaling and promotes new vessel wall remodeling^[11]^. ENG overexpression also protects against TGFβ-induced apoptosis of endothelial cells^[17, 59]^. Reduction of vascular cell-apoptosis after hypoxia improves blood supply to ischemic tissue^[58, 71]^. Increase of ENG expression in endothelial cells could also be hazardous, because BBB permeability was increased in some of the capillaries that express high level of ENG, which was accompanied with mononuclear cell infiltration in the surrounding brain tissues^[72]^. These findings suggests that pronounced ENG overexpression might impair vessel wall integrity. Alternatively, lack of ENG expression may indicate severe vessel damage^[72]^. ENG and TGFβ are involved in the pathogenesis of post-ischemic brain injury in human. Abnormal ENG and TGFβfunction might lead to long-term neurological deterioration or cognitive disturbance after acute ischemic stroke^[72, 73]^. Homeostasis of ENG expression is crucial for maintaining normal angiogenesis, vascular remodeling and reduction of stoke injury.

## THE EFFECT OF ENG DEFICIENCY IN ISCHEMIC STROKE INJURY

The survival of neurons in peri-infarcted regions is associated with the extent of patient recovery after stroke^[74]^. Nutrient supply supporting neuron survival is carried through blood. Higher microvessel density in the peri-infarct region is associated with lower morbidity and mortality^[22]^. Hypoxia-induced angiogenesis increases blood flow and oxygen delivery to ischemic tissues, which contributes to the recovery after stroke^[28]^.

Angiogenesis occurs in human brain after stroke. Through examining human postmortem brain samples with ischemic infarcts caused by occlusive vascular diseases, capillary networks with regular connection and micro-vessels were found in the brain samples of patients who died within one week after stroke, and the neo-vasculature was in filled with blood in the brain samples collected from patients that died 2–3 weeks after stroke^[75]^. The micro-vessel density remains higher in the infarct area compared with the corresponding contralateral side three months after stroke^[22]^. Increased vessel density restores cerebral blood flow, salvages ischemic tissue, enhances neuronal survival and improves functional recovery of stroke survivors^[76]^.

ENG is expressed in proliferating vascular endothelial cells^[77]^ and is elevated in inflammatory tissue and healing wound^[78]^. In patients, somepro-angiogenic genes, including Tie-2, matrix metalloproteinase-2 (MMP-2), tissue inhibitor of matrix metalloproteinase-1 (TIMP-1), hepatocyte growth factor-α (HGF-α) and monocyte chemoattractant protein-1 (MCP-1), were upregulated in ENG expressing micro-vessels in stroke affected tissue. These key angiogenic elements play important roles in endothelial cell migration, differentiation and tube-formation, as well as vessel stabilization and stem cell homing into the region of angiogenesis and revascularization^[79]^.

In *Eng* deficient mice (*Eng*^+/−^ mice), the functional performance after stroke was poorer than wild type animal both in the acute phase and the sub-chronic stage (one month after stroke), suggesting that there is an association between delayed functional recovery and *Eng* deficiency^[50]^. The infarct volume and atrophic volume are larger in *Eng*^+/−^ mice^[50]^. The density of micro-vessels within the infarct and peri-infarct region are lower in *Eng*^+/−^ mice than wild type mice^[50, 80]^. *In vitro* study showed that *Eng*^+/−^ endothelial cells express a lower level of VEGF^[81]^ compared to that of wild type endothelial cells. *Eng*^+/−^ macrophages express lower levels of VEGF receptor 1 (VEGFR1) and 2 (VEGFR2) at the baseline and lower level of VEGFR2 after VEGF stimulation than wild type macrophages^[42]^. Although *Eng*^+/−^ macrophages and wild type macrophages express similar levels of MMP9 at the baseline, unlike in wild type macrophages, the expression of MMP9 did not increase in *Eng*^+/−^ macrophages after VEGF treatment^[42]^. In the brain of *Eng*^+/−^ mice, VEGF-induced upregulation of VEGFR2 expression was also impaired^[82]^. Together, these data suggest a reduced angiogenic response in the absence of normal *Eng* function may be responsible for the impairment of tissue repair in *Eng* deficient mice after experimental stroke.

In addition, our study suggested that *Eng* deficiency is associated with impairment of macrophage recruitment and clearance in the peri-infarct area during stroke recovery^[50]^. Eng expression was upregulated during the transition from monocyte to macrophage^[7]^. *Eng* deficiency in endothelial cell reduced adhesion and transmigration of leukocytes in response to ischemic injury^[83]^. Recruitment of monocytes to the infarcted tissue and subsequent vessel formation was severely impaired in HHT1 patients (who have *ENG* haploinsufficiency)^[80]^ suggesting that *ENG* deficiency impairs monocyte adhesion and migration. In the acute phase (3 days) of stroke, *Eng* deficient mice had fewer macrophages in the peri-infarct region^[50]^. However, at 60 days after stroke, a time that is considered as recovery stage, there was an increase number of macrophage in the peri-infarct region of *Eng* deficient animals^[50]^, suggesting a delayed homing and clearance of over-activated macrophage. However, the roles of post-ischemic inflammation might be bidirectional^[84]^. The inflammatory response after ischemic stroke could contribute to a secondary brain injury, because the influx of inflammatory cells amplifies brain cell death. On the other hand, inflammation also facilitated the clearance of damaged tissues and promoted tissue repair^[85]^. Therefore, the consequences of impaired macrophage in homing and clearance in the stroke tissue require further studied.

Interestingly, *Eng*^+/−^ mice had severer brain injury than wild type mice since the first day of experimental stroke^[50]^, which could not be explained by impaired tissue repair. As discussed in above, hypoxia induce endothelial *Eng* expression, which prevents hypoxia-induced apoptosis of endothelial cells. Therefore, vascular damage in *Eng*^+/−^ mice could be more severe than in wild type mice after ischemic injury. In addition, *Eng* haploinsufficiency has been shown to be associated with reduced production of nitric oxide and increased production of superoxide under eNOS induction^[86]^. Nitric oxide produced by endothelial cell induces vascular relaxation^[87]^. Bioavailability of nitric oxide is lower in *Eng*^+/−^ mice than in wild type mice^[88]^. Enhancing superoxide production in *Eng* deficient mice reduces vascular relaxation, and increases vessel damage and oxidative stress, all of which increases brain injury during the acute stage of ischemic stroke.

Since ENG plays an important role in angiogenesis and lack of ENG dampens angiogenesis, therapeutic stimulation of ENG could promote angiogenesis, vascular remodeling and improve stroke recovery, as well as reduce morbidity and mortality of stroke patients.

## CIRCULATING SOLUBLE ENG MODULATES CEREBRAL VASCULAR REMODELING AND PLAYS ROLES IN VASOSPASM AFTER SUBARACHNOID HEMORRHAGE

Soluble ENG (sENG) is an alternative transcript of *ENG* gene, which contains only the extracellular domain of the full-length ENG. Soluble ENG enters the circulation in various conditions that related to the endothelial injury, activation, inflammation and senescence^[89]^. Our group showed that sENG level is increased in the surgical resected human bAVMs^[90]^. We have also shown that co-injection of an adenoviral vector expressing sENG with AAV-VEGF into mouse brains caused capillary dysplasia. It is still unclear how overexpression of sENG causes cerebrovascular malformation. One of the possibilities is that circulating sENG acts as a decoy inhibiting the effect of ENG on the endothelium, leading to vascular malformation during angiogenesis.

Nitric oxide (NO) is a potent vascular smooth muscle relaxant, which is synthesized by the vascular endothelium. *Eng*^+/−^ mice have a lower level of NO metabolites (nitrites) in the plasma and in the urine than that of wild type mice^[91]^, suggesting that the NO level is lower in *Eng* deficient animals. The hypotensive and vasodilatory response induced by endothelium-dependent vasodilators was less intensive in *Eng*^+/−^ mice than wild type mice. However, the difference of this vasodilation effect between *Eng*^+/−^ mice and wild type mice disappeared after NO synthesis was inhibited^[91]^. These findings suggested that the NO level or the subsequent vessel response to NO is reduced in *Eng*^+/−^ mice. However, after eliminating the endogenous NO, the vasodilatory effect induced by exogenous NO donor (nitroprusside) was similar in *Eng*^+/−^ and wild type mice^[91]^. The peripheral progenitor cells of HHT patients expresses lower level of eNOS (endothelial nitric oxide synthase) mRNA^[92]^. Endothelial NOS produces NO in response to humoral and mechanical stimuli. However, resistance arteries in *Eng*^+/−^ mice displayed an eNOS-dependent impairment in the myogenic response (normal resistance arteries contract in response to increases of perfusion pressure) despite of a reduced eNOS level. *Eng* deficient endothelial cells had uncoupled eNOS, which produce less NO but more superoxide^[86]^. Taken together, these studies indicate a role of *Eng* in the regulation of vascular tone.

Cerebral vasospasm is one of the most common complications of subarachnoid hemorrhage (SAH) and is associated with high morbidity and mortality. NO is found to be an important mediator of vasospasm^[93]^. The potential role of ENG on the production of NO suggests that ENG might be associated with vasospasm after SAH. In patients with SAH, the level of sENG increased in the cerebrospinal fluid (CSF) and decreased in the serum^[94, 95]^. In the subgroup with cerebral infarction due to post-SAH vasospasm, the level of sENG was higher in the CSF and lower in the serum than the patients who did not have post-SAH cerebral infarction^[94, 95]^. The level of sENG during the first two weeks of SAH might be a predictive factor for the long-term outcome, such as, 6 months after SAH^[95]^. Similar to sENG, the ENG positive endothelial micro-particles were increased in SAH patients with vasospasm^[96]^.

Soluble ENG are present in both healthy people and patients with pathological conditions (such as preeclampsia and SAH)^[89]^. Several studies suggest that sENG is a naturally occurring antagonist of TGFβ^[97]^. In contrast to the lower level of sENG, the level of TGFβ1 in the serum was higher in patients with vasospasm after SAH than those without vasospasm^[95]^. Moreover, sENG interferes the binding between TGFβ1 and its receptors^[89]^. TGFβ1 has been suggested to be involved in eNOS activation^[89]^. Therefore, the reduced sENG levels in patients with post-SAH vasospasm might reflect an impaired production of vasorelaxant factors, such as NO. However, there is no direct evidence supporting the cause-and-effect relationship between vasospasm and sENG. Further studies of post-SAH vasospasm in *Eng*-deficient mice might be helpful in exploring the association of sENG and vasorelaxation.

Interestingly, the changes of sENG in the cerebrospinal fluid (CSF) and the serum of patients with SAH and vasospasm are opposite^[94, 95]^. In patients with Doppler sonographic vasospasm, the serum level of sENG was similar to those without vasospasm^[95]^. However, the serum level of sENG was reduced in patients with cerebral infarction due to severe vasospasm and hydrocephalus^[95]^, suggesting that the sENG level in the serum might beserved as a biomarker for cerebral ischemia subsequent to vasospasm. Cerebral hypo-perfusion or hypoxia could induce increases of focal expression of ENG and might contribute to the increase of sENG in the CSF of patients with vasospasm. Both extravasation of sENG from blood or intrathecal production of sENG could cause the increase of sENG in the CSF and decrease of sENG in the plasma. Further studies are needed to ravel the origin of sENG during post-SAH vasospasm.

Although it is not clear how ENG-positive micro-particles and sENG increased in patients with post-SAH vasospasm, the results of these studies indicated that, the circulating sENG is a promising biomarker for cerebral vasospasm after SAH.

## ALTERNATIONS OF ENG EXPRESSION IN ATHEROSCLEROTIC PLAQUES AND STENOTIC CEREBRAL VESSELS

Carotid atherosclerotic stenosis is a major cause of ischemic stroke. As mentioned in earlier sections, ENG is expressed mainly in endothelial cells, smooth muscle cells and macrophages, which are the three major cells involved in the pathogenesis of atherosclerosis^[10]^. The expression of ENG is very low in normal human arteries and is restricted to the endothelial cells of adventitial microvessels^[10]^. In contrast, higher ENG expression is present in the advanced atherosclerotic plaque of human patients^[10]^. The site of ENG expression are slightly difference between atherosclerotic plaques in carotid arteries and aorta. In aortic atherosclerotic plaque, ENG is predominantly expressed in smooth muscle cells. However, in carotid plaque, ENG is expressed in endothelial cells of neo-vessels within the lipid core and plaque shoulders^[98]^. ENG expression is higher in carotid plaque containing higher levels of collagen and lessintra-plaque thrombi, which are characteristics of stable plaques^[99]^. These evidence indicate that ENG may promote the formation of intra-plaque neo-vessels and collagen and reduce the vessel leakage and hemorrhage. However, ENG expression in the neo-vessels of carotid atherosclerosis has also been found to be positively correlated with the advanced grade of plaques^[100]^. The distinct ENG expression patterns in different types of plaques suggest that ENG might play different roles in the course of atherogenesis progression.

Atorvastatin is a drug to treat carotid atherosclerotic plague. In a mouse model of atherosclerosis, atorvastatin treatment decreased the level of Eng in the serum and increased Eng expression in the plaque^[101]^. Therefore, Eng may serve as a biomarker for evaluating the therapeutic effect of drugs in treating atherosclerosis. More studies are needed to elucidate the role of ENG in the pathogenesis of atherosclerosis.

Moyamoya disease (MMD) is a rare, progressive cerebrovascular disorder caused by blocked arteries at the base of the brain in an area called the basal ganglia. MMD is one of the major causes of stroke in children and adults characterized by progressive stenosis or occlusion of terminal portion of internal carotid arteries and development of fragile collateral vessels^[102]^. Middle cerebral artery (MCA) of MMD patients had thicker intimal walls than control vessels collected from aneurysm patients^[103]^, indicating intimal hyperplasia in MMD. The expression of ENG and HIF-1 are increased in the intima of MMD patients^[103]^. In addition, TGFβ3 expression was also detected, which was predominantly in the endothelium and was co-localized with HIF-1 and ENG^[103]^. Although the study did not find an association between cerebral blood perfusion and ENG expression^[103]^, the low spatial resolution method used to evaluate the cerebral blood flow of the entire MCA territory might not be accurate enough to detect the real perfusion through the MCA branches that were used to measure the ENG expression. The increased expression of ENG and HIF-1 in MMD is consistent with the increased expression of ENG under hypoxia condition. Therefore, ENG may play roles in the pathogenesis of cerebrovascular stenosis or occlusion.

## THE PROSPECTIVE OF MODULATING ENG EXPRESSION FOR THE TREATMENT OFCEREBROVASCULAR DISEASES

Since ENG has been implicated in the pathogenesis of various cerebrovascular diseases [Table 2], modulation of ENG expression might be a potential treatment for these conditions. Although currently there is no treatment available for patients with human cerebrovascular diseases through targeting ENG, several agents that affect ENG expression specifically or non-specifically, are clinical available for treating patients or are used in clinical trials. TRC105 is a chimeric IgG1monoclonal antibody specifically against ENG that inhibits angiogenesis, induces antibody-dependent cellular cytotoxicity (ADCC) and apoptosis of proliferating endothelium. The safety and activity of TRC105 have been tested in a Phase I and a preliminary Phase II clinical trials in cancer patients^[104]^. Resveratrol is a natural component of a number of fruits, including grapes, blueberries and raspberries. The skin of red grapes is used to extract resveratrol. *In vitro*, resveratrol reduces sENG secretion and pro-inflammatory factors of cultured endothelial cells^[105]^. Therefore, it might be a promising non-specific inhibitor of sENG. A proper level of ENG expression might be crucial for maintaining normal angiogenesis and vascular remodeling in the brain. However, there is no report of direct regulation of ENG for treating cerebrovascular disease to date. *In vitro* study showed that statins could increase sENG secretion from endothelial cells^[106]^; and *in vivo* administration of stain increased Eng expression in the carotid plaque of a mouse model^[101]^. Statins are a group of medications that has been used to treat patients with carotid artery atherosclerosis and other ischemic cerebrovascular diseases. More studies are needed to test whether ENG can be used as a target for developing new therapies for the treatment of cerebrovascular diseases.

## CONCLUSION

In summary, ENG plays a critical role in angiogenesis, vascular development and regulation of vascular tone. ENG deficiency is associated with the development of AVM in HHT patients, exacerbates stoke injury and impairs stroke recovery. ENG might be a potential biomarker for vasospasm after SAH and cerebrovascular stenosis. Therefore, experimental or therapeutic modulating of ENG expression are useful ingeneration of disease models in animals to study disease pathogenesis and in development of novel therapies to treat cerebrovascular diseases. The exact function of ENG in cerebrovascular diseases remains to be revealing.

## Figures and Tables

**Table 1:** Summary of ENG expression patterns in tissue and cell lines

Tissue	Tissue samples	Cell lines
Brain	Humanendothelium^[13]^	
	Human adventitia^[13]^	
Non-brain	Human placenta^[6]^	HUVEC^[5]^
	Human spleen^[7]^	HOON^[6]^
	Murine ovary and uterus^[8]^	U-937^[6, 7]^
	Murine heart^[8]^	HL- 60 ^[7]^
	Murine muscle^[8]^	Cultured monocytes^[7]^
	Murine placenta^[8]^	NCTC-2071^[8]^
	Murine spleen^[8]^	VSMC^[9]^
		HASMC^[10]^

**Table 2:** Cerebrovascular diseases and ENG level

Cerebrovascular diseases	Species	Specimen	ENG level	Clinical or biological observation	Author and year
Stroke	Human	Brain	Increased	-	Krupinski *et al*., ^[22]^ 1994
	Human	Serum	Increased	Positive correlation with stroke severity	Kim *et al*., ^[69]^ 2012; Simak *et al*., ^[70]^ 2006
	*Eng*^+/−^ mouse	Brain	Decreased	Poorer functional performance, larger infarction, less angiogenesis, impaired macrophage recruitment and clearance	Shen *et al*., ^[50]^ 2014
Brain AVM	*Eng*^+/−^ mouse	Brain	Decreased	Cerebrovascular dysplasia after VEGF stimulation	Hao *et al*., ^[37, 40]^ 2010
	*Eng*^*2f*/*2f*^ mouse	Brain	Decreased after gene KO	Brain AVM after VEGF stimulation and *Eng* KO	Choi *et al*., ^[46]^ 2012; Choi *et al*., ^[47]^ 2014
	HHT1 patient	Somatic cell	Decreased	Higher incidence of AVM in brain and pulmonary	McAllister *et al*., ^[24]^ 1995
Vasospasm SAH	Human patient	Serum	Decreased	patients with cerebral infarction,	Dietmann *et al*., ^[95]^ 2012
	Human patient	CSF	Increased	patients with cerebral infarction	Testai *et al*., ^[94]^ 2011
Carotid stenosis	Human patient	Carotid plaque	Increased	Positive correlation with stage of plaque	Conley *et al*., ^[10]^ 2000; Bot *et al*., ^[99]^ 2009; Luque *et al*., ^[100]^ 2009
	Mouse	Carotid plaque	Increased	Atrovastatin increase *Eng* expression	Rathouska *et al*., ^[101]^ 2011
Moyamoya disease	Human patient	Intima of MCA	Increased	-	Takagi *et al*., ^[103]^ 2007

AVM: arteriovenous malformation; CSF: cerebrospinal fluid; HHT: hereditary hemorrhagic telangiectasia; MCA: middle cerebral artery; KO: knock-out; SAH: subarachnoid hemorrhage; VEGF: vascular endothelial growth factor
